# Sleep Patterns Fluctuate Following Training and Games across the Season in a Semi-Professional, Female Basketball Team

**DOI:** 10.3390/brainsci13020238

**Published:** 2023-01-31

**Authors:** Cody J. Power, Jordan L. Fox, Masaru Teramoto, Aaron T. Scanlan

**Affiliations:** 1 School of Health, Medical and Applied Sciences, Central Queensland University, Rockhampton, QLD 4701, Australia; 2 Rural Clinical School, The University of Queensland, Rockhampton, QLD 4700, Australia; 3 Department of Physical Medicine and Rehabilitation, University of Utah, Salt Lake City, UT 84108, USA

**Keywords:** recovery, diurnal, circadian, behavior, team sport, women

## Abstract

Quantifying athlete sleep patterns may inform development of optimal training schedules and sleep strategies, considering the competitive challenges faced across the season. Therefore, this study comprehensively quantified the sleep patterns of a female basketball team and examined variations in sleep between nights. Seven semi-professional, female basketball players had their sleep monitored using wrist-worn activity monitors and perceptual ratings during a 13-week in-season. Sleep variables were compared between different nights (control nights, training nights, training nights before games, nights before games, non-congested game nights, and congested game nights), using generalized linear mixed models, as well as Cohen’s *d* and odds ratios as effect sizes. Players experienced less sleep on training nights before games compared to control nights, training nights, nights before games, and congested game nights (*p* < 0.05, *d* = 0.43–0.69). Players also exhibited later sleep onset times on non-congested game nights compared to control nights (*p* = 0.01, *d* = 0.68), and earlier sleep offset times following training nights before games compared to all other nights (*p* < 0.01, *d* = 0.74–0.79). Moreover, the odds of players attaining better perceived sleep quality was 88% lower on congested game nights than on nights before games (*p* < 0.001). While players in this study attained an adequate sleep duration (7.3 ± 0.3 h) and efficiency (85 ± 2%) on average across the in-season, they were susceptible to poor sleep on training nights before games and following games. Although limited to a team-based case series design, these findings suggest basketball coaches may need to reconsider scheduling team-based, on-court training sessions on nights prior to games and consider implementing suitable psychological and recovery strategies around games to optimize player sleep.

## 1. Introduction

Sleep is considered essential for optimal health, performance, and recovery in sport [1]. Less than the recommended amount of sleep (<7 h of high-quality sleep per night for adults) [2] has been shown to negatively affect athletic performance outcomes [3], such as reaction time [4] and maximal oxygen consumption [5], as well as recovery-related outcomes, including muscle protein synthesis [6] and nightly cortisol concentrations [7]. In addition, sleep disturbances (e.g., poor sleep quality) can increase daytime fatigue, which may impact training quality in team-sport athletes [8]. In this regard, team-sport athletes appear susceptible to inadequate and low-quality sleep across the season, given that they may regularly participate in congested game schedules (i.e., multiple games played in the week or round of competition), undertake short- or long-haul travel [9], and are exposed to high stress scenarios (i.e., competition) [8]. Consequently, many researchers have quantified the sleep patterns of athletes from various team sports to identify sleep-related issues that can be subsequently addressed to optimize athlete health, performance, and recovery [10]. 

Regarding sleep patterns among team sport athletes, reviews highlight that most studies on this topic have recruited football players (i.e., soccer, Australian rules football, rugby league, rugby union, American football), with little research attention dedicated to basketball players (proportion of studies in review examining football vs. basketball players: 63% vs. 6% [11] and 49% vs. 4% [12]). Given that basketball players have been shown to sleep longer [13,14], wake later [13,14], spend more time awake after sleep onset [14], and take longer to go to sleep [14] than football players [13,14], the bulk of existing sleep data reported in team-sport athletes cannot be simply transferred from football players to basketball players. Consequently, targeted sleep data are needed to understand the sleep patterns of basketball players and identify any sleep-related issues in this population. 

When quantifying sleep in basketball players, it is important to identify how sleep patterns are affected by training sessions and games to best inform optimal implementation of training schedules and sleep strategies across the season. In this way, sleep duration and quality may be disrupted following training sessions and games conducted at night, due to elevated mental stimulation [15], cognitive fatigue [15], and muscle soreness [9]. Moreover, evening games may promote later bedtimes among players, due to the requirement to complete post-game commitments, such as interviews, recovery regimes, and social functions [16]. The potential for competition to negatively impact subsequent sleep patterns in players may also be amplified when congested game schedules are faced, given that they augment the accumulative muscle damage experienced [17,18]. Muscle damage is likely to increase perceived muscle soreness and thereby disrupt sleep [19]. Consequently, the potential impact of training and game scheduling on sleep patterns emphasize the need to examine basketball players longitudinally across the season to adequately account for these factors. 

While some studies have examined the longitudinal sleep patterns of basketball players, they have primarily examined male basketball players [20,21,22,23,24]. Growing research indicates that sleep data collected in male athletes should not be simply applied to female athletes, due to sex differences in sleep duration and sleep quality [12,25,26], which may relate to chronobiology [27,28] and psychological variations [29,30]. In addition, there is an elevated risk for, and prevalence of, several sleep disorders in females compared to males (i.e., restless leg syndrome and insomnia) [29,31]. In this way, only four studies have monitored the sleep patterns of female basketball players longitudinally across the season. Specifically, Taber et al. [32] reported that first-division collegiate players (*n* = 16) attained 6.7 ± 0.9 h of sleep per night, measured using a wrist-worn device (WHOOP; Boston, MA, USA) across 3 weeks of the off-season and 6 weeks of the pre-season combined. In turn, Dunican et al. [33] documented that professional players (*n* = 12) competing in the Australian Women’s National Basketball League (WNBL) attained 8.1 ± 1.6 h of sleep at an efficiency (i.e., measure of sleep quality whereby sleep duration is expressed as a percentage of time in bed) of 92 ± 5% per night, measured using wrist-worn activity monitors (Readiband^TM^; Fatigue Science Inc, Vancouver, Canada) during 4 weeks of the in-season, before undergoing an educational intervention. Considering game schedules, Staunton et al. [34] observed that WNBL players (*n* = 17) attained 7.6 ± 1.5 h of sleep at 92 ± 4% efficiency per night across two consecutive in-seasons using wrist-worn activity monitors (GT3-X; ActiGraph, Pensacola, FL, USA), but experienced significant (*p* < 0.05) reductions in sleep duration on nights following the second game in double-headers (i.e., two games in one week) compared to nights following games in single-game weeks [34]. More recently, Miles et al. [14] reported that WNBL players (*n* = 11) attained 7.4 ± 1.5 h of sleep at 88 ± 6% efficiency (training days and rest days combined) using wrist-worn activity monitors (GTX3+; ActiGraph, Pensacola, FL, USA) across 4 × 1-week blocks of the pre-season and in-season. Comparisons between nights before games, the night of games, and nights following game day showed players had a significantly (*p* < 0.05) higher wake after sleep onset (time spent awake following sleep onset) on the night before a game than the night of games [14]. Collectively, these studies show that female basketball players may attain adequate (>7 h) sleep durations and quality (efficiency ≥ 85%) [35]; however, some notable gaps in evidence are apparent. 

First, no data are available detailing the sleep patterns of semi-professional, female basketball players. Unlike professional basketball players, semi-professional players are given less remuneration and, as such, likely have further work or study commitments that may impact their sleep [36]. Second, only one study [34] has examined the impact of congested game schedules on sleep in female basketball players from a single professional basketball team; therefore, additional studies on this topic are needed. Finally, no study has compared sleep between control nights, training nights, and nights surrounding games. Therefore, to address these gaps, this study aimed to comprehensively quantify the sleep patterns of semi-professional, female basketball players across the season, and examine variations between control, training, non-congested game (i.e., one game played in the week or round of competition), and congested game nights.

## 2. Methods

### 2.1. Subjects

Ten players volunteered to participate in this study and were monitored across the season. However, due to non-participation in games (<2 min of playing time on average per game), two players were excluded from the analyses. Another player was excluded from analyses due to a lack of compliance in wearing the wrist-worn activity monitor. Therefore, seven semi-professional, female basketball players (age 20 ± 2 years, stature 178 ± 8 cm, body mass 74 ± 14 kg) contributed sleep data for analyses in this study. All players were from the same team registered in the NBL1 North Conference, which is a second-tier Australian basketball competition held at the state level. Players were informed of the risks and benefits of participation; they gave verbal and written informed consent prior to study commencement. All players completed the Adult Pre-Exercise Screening System to ensure they were free from any injuries or health conditions preventing safe participation. Given the observational nature of the study, players were encouraged to continue their usual dietary, training, and sleep habits, with no feedback provided regarding sleep data across the season, to avoid influencing player behaviors [1]. During the screening process, all players indicated that they had not previously been diagnosed with a sleep disorder and did not currently have one. Ethical approval for this study was received from the Central Queensland University Human Research Ethics Committee (number: 0000023323).

### 2.2. Design

**Figure 1:**
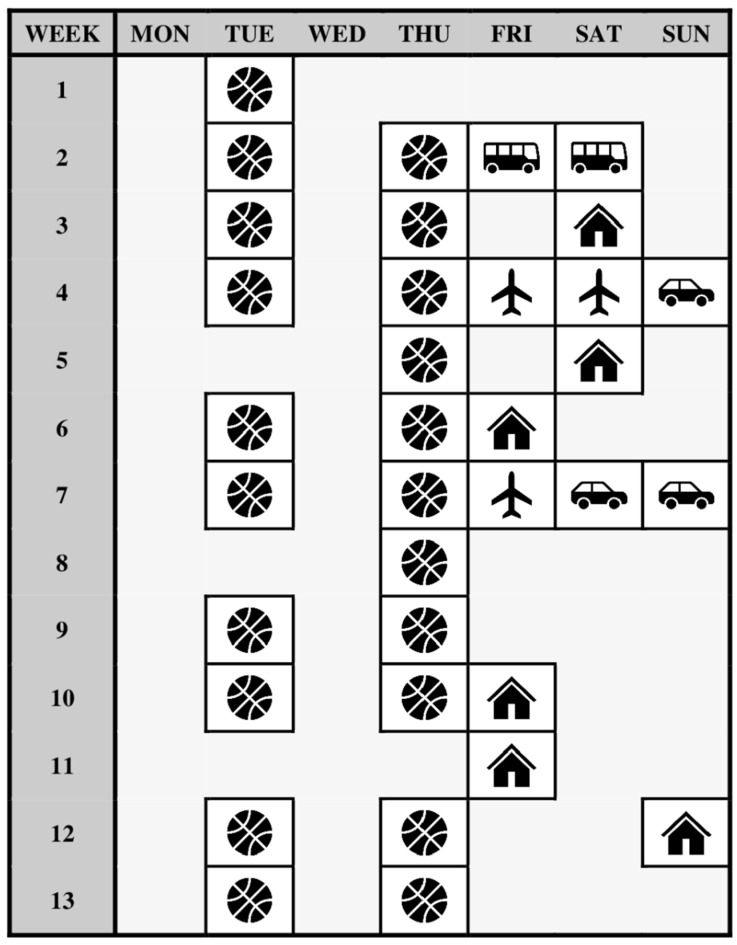
The training, travel, and game schedule of the semi-professional, female basketball team monitored in this study. Note: Empty shaded cell indicates control night; 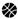 indicates on-court team training session; 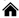 indicates home game; 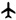 indicates away game that was travelled to by plane; 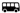 indicates away game that was travelled to by bus; 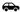 indicates away game that was travelled to by car.

Given the longitudinal, observational study design adopted, the Strengthening the Reporting of Observational Studies in Epidemiology (STROBE) statement was followed [37]. Player sleep patterns were monitored across the 2021 in-season phase, with the full team training, travel, and game schedule for the season shown in Figure 1. The in-season phase was shortened by 4 weeks compared to the typical in-season duration, due to restrictions stemming from the Coronavirus disease (COVID-19) pandemic. All training sessions were held from 18:30 to 20:00, all night games (played on Fridays and Saturdays) were held between 18:00 and 21:00, and all day games (played on Sundays) were held between 12:00 and 15:00. Across the study, the compliance rate for wearing sleep monitors across all nights was 85 ± 16% (range = 51–97%), while the compliance for completing online perceptual sleep ratings each day was 60 ± 28% (range = 26–91%).

### 2.3. Procedures

Prior to study commencement, anthropometric data were collected on each player, following established protocols [38]. Stature was measured using a portable stadiometer (Seca 213; Seca GmbH, Hamburg, Germany), while body mass was measured using electronic scales (BWB-600; Tanita Corp, Tokyo, Japan) with players removing shoes and wearing normal training attire for measurement. Sleep patterns were monitored each night using wrist-worn activity monitors (Readiband™, Fatigue Science, Vancouver, BC, Canada) as per established recommendations [39]. Players were instructed to always wear the activity monitor on the non-dominant wrist, and to remove it when participating in training and games, or when swimming and showering. Readibands™ use a 16-Hz tri-axial accelerometer to estimate time spent awake and asleep, dependent upon the activity levels detected, and are configured to collect data in 1-s epochs. 

Players also recorded their perceived sleep quality each morning upon awakening, using an online application (Team App, www.teamapp.com) on their personal smartphone. Perceived sleep quality was measured in arbitrary units (AU) using a 5-point Likert-type scale, in line with previous studies [21,40], whereby 1 = very poor sleep quality, 2 = poor sleep quality, 3 = average sleep quality, 4 = good sleep quality, and 5 = very good sleep quality. All perceived sleep quality data were collated using a secure online platform (Google Forms, www.docs.google.com/forms).

Once collected, all sleep data were extracted and organized into a custom Microsoft Excel spreadsheet (version 16; Microsoft Corporation, Redmond, WA, USA). All sleep variables measured in this study are defined in Table 1. The inter-device reliability (intra-class coefficient = 0.80–0.99) [41] and validity (sensitivity = 94%, specificity = 40%, and accuracy = 90%) in detecting sleep and wake compared to polysomnography (PSG) [42], has been previously documented for Readibands™. Moreover, Readibands™ have been demonstrated to gather comparable (*p* > 0.05) measures of total sleep time, wake after sleep onset, and sleep efficiency, compared to PSG [42,43]. To examine how player sleep patterns fluctuate around training and games, sleep variables were grouped into the following night types:

- Control nights—no training or game were performed on that day and no game was played on the following day.
- Training nights—team-based, on-court training sessions were completed earlier that evening and no game was played on the following day.
- Training nights before games—team-based, on-court training sessions were completed earlier that evening and a game was played on the following day.
- Nights before games—no team-based, on-court training was completed throughout that day and a game was played on the following day.
- Non-congested game nights—a game was played during that day which was either the only game played that week or the first game of a congested game schedule (i.e., two or three games played on successive days).
- Congested game nights—a game was played during that day which was the second or third game of a congested game schedule.

### 2.4. Statistical Analysis

A generalized linear mixed model (GLMM) [44,45] was fit to the data on each sleep variable (except perceived sleep quality), using players as a random effect and night type as fixed effects (reference category = control) for model-building and initial comparison. In turn, Gaussian family and identity links with unstructured variance-covariance structures were chosen to build the models. A histogram inspection revealed that some sleep variables, including wake after sleep onset and efficiency, were not normally distributed, potentially resulting in heteroscedasticity of residuals, along with a few outlying cases. Consequently, each GLMM was built by computing the robust estimate of variance (Huber-White sandwich estimator) that can account for outliers and heteroscedasticity of residuals to obtain robust models [46,47,48]. As this study was exploratory in nature, all pairwise comparisons were performed as post hoc analyses to identify significant differences in sleep variables between night types using the Scheffe method [49]. Marginal means ± standard errors (SE) were also calculated for each sleep variable (except perceived sleep quality) for each night type. An alpha level was set at 0.05 to indicate statistical significance. Further, Cohen’s *d* effect sizes were calculated for each pairwise comparison, using an unstandardized regression coefficient and pooled standard deviation [50]. The magnitude of *d* was interpreted as: *small* = 0.20–0.49; *medium* = 0.50–0.79; and *large* = ≥0.80 [51]. Meanwhile, given the ordinal nature of the perceived sleep quality data, player data were summarized using frequency counts and percentages for each night type. In turn, perceived sleep quality was fit using a GLMM with an ordinal family and logit link, along with an unstructured variance-covariance structure and robust estimate of variance. Specifically, a series of models were built, using each of the six night types, one by one, as a reference category. An odds ratio (OR) with 95% confidence intervals (CI) was calculated for each model. The Bonferroni correction was used to adjust the alpha level to control for type I error rates. The OR was used as an effect size indicator. All analyses were conducted using Stata/MP (version 17.0 for Windows; StataCorp LLC; College Station, TX, USA).

## 3. Results

**Figure 2:**
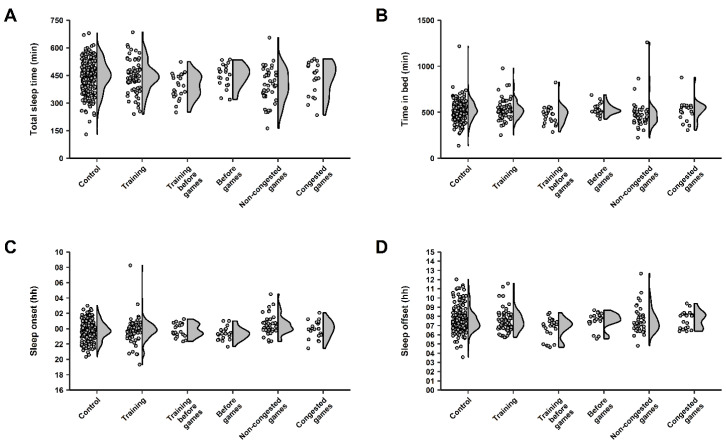
Individual data points (dot plots) and data dispersion (half-violin plots) for (**A**) total sleep time, (**B**) time in bed, (**C**) sleep onset, and (**D**) sleep offset in the semi-professional, female basketball players monitored across the in-season in this study.

**Figure 3:**
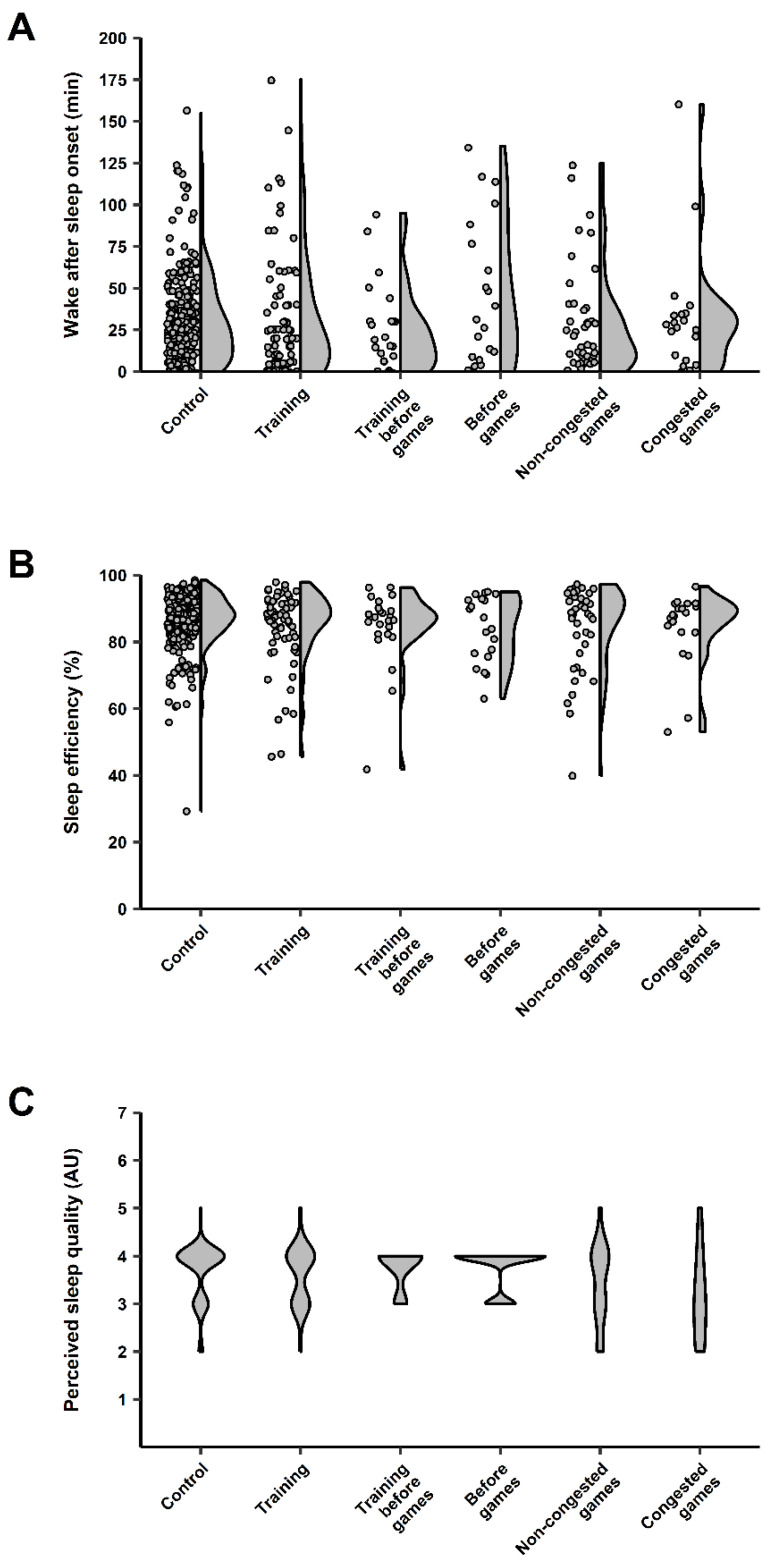
Individual data points (dot plots) and data dispersion (half-violin plots) for (**A**) wake after sleep onset (WASO), (**B**) sleep efficiency, and (**C**) perceived sleep quality in the semi-professional, female basketball players monitored across the in-season in this study. Note: Perceived sleep quality is presented using full violin plots, given that these data were measured using a 5-point Likert-type scale, and, therefore, individual data points could not be effectively presented graphically.

Players were monitored for 91 nights across the in-season, resulting in 408 data points, which included: 52 control nights (232 data points), 21 training nights (71 data points), 5 training nights before games (24 data points), 4 nights before games (20 data points), 9 non-congested game nights (41 data points), and 5 congested game nights (20 data points). Marginal means ± SE, along with *p* values from GLMM analyses, for all sleep variables (except perceived sleep quality) are shown in Table 2. Furthermore, the individual samples and data dispersion for each sleep variable are shown in Figure 2 (total sleep time, time in bed, sleep onset, and sleep offset) and Figure 3 (wake after sleep onset, efficiency, and perceived sleep quality).

A significant main effect was observed between night types for total sleep time (*p* < 0.001). Post hoc analyses showed players experienced significantly less total sleep time on training nights before games compared to control nights (*p* = 0.02, *d* = 0.68, *medium*), training nights (*p* < 0.001, *d* = 0.68, *medium*), nights before games (*p* = 0.002, 0.69, *medium*), and congested game nights (*p* = 0.02, *d* = 0.43, *small*). Although non-significant (*p* >0.05), *medium* effects were reached in other comparisons, whereby players achieved less total sleep time on non-congested game nights compared to control nights (*d* = 0.61), training nights (*d* = 0.61), and nights before games (*d* = 0.60). 

No significant main effect was observed between night types for wake after sleep onset (*p* = 0.15). In turn, *medium* effects were reached where more wake after sleep onset was experienced by players on nights before games than control nights (*d* = 0.52), training nights before games (*d* = 0.67), and non-congested game nights (*d* = 0.54). In contrast, a significant main effect was observed between night types for time in bed (*p* < 0.001). Post hoc analyses revealed players spent significantly less time in bed on training nights before games than nights before games (*p* < 0.001, *d* = 0.54, *medium*). Although non-significant (*p* > 0.05), players also spent less time in bed on training nights before games than training nights (*d* = 0.54, *medium*). While a significant main effect between night types was observed for efficiency (*p* < 0.001), post hoc analyses did not reveal any significant pairwise comparisons nor comparisons reaching a *medium* effect.

A significant main effect was found between night types for sleep onset (*p* < 0.001). Post hoc analyses showed players experienced a later sleep onset on non-congested game nights compared to control nights (*p* = 0.01, *d* = 0.68, *medium*). Further, non-significant, *medium* pairwise differences were also observed, whereby players had a later sleep onset after non-congested games compared to training nights (*d* = 0.63), training nights before games (*d* = 0.61), and congested game nights (*d* = 0.56). A significant main effect was also observed between night types for sleep offset (*p* < 0.001). Post hoc analyses revealed players woke significantly earlier on mornings following training nights before games compared to mornings following control nights (*p* < 0.001, *d* = 0.76, *medium*), training nights (*p* = 0.003, *d* = 0.76, *medium*), nights before games (*p* = 0.007, *d* = 0.79, *medium*), non-congested game nights (*p* = 0.007, *d* = 0.77, *medium*), and congested game nights (*p* = 0.002, *d* = 0.74, *medium*). Statistics for all pairwise comparisons for each sleep variable (except perceived sleep quality) are provided in Supplementary Table S1.

For perceived sleep quality, the frequency counts and percentages for each perceptual rating, using the 5-point Likert-type scale for each night type, are shown in Table 3. Furthermore, the OR with 95% CI quantifying the odds of attaining higher perceived sleep quality across different night types are shown in Table 4. Analyses revealed that the odds of players attaining better perceived sleep quality was 88% lower on congested game nights than on nights before games (OR [95% CI] = 0.12 [0.05, 0.30], *p* < 0.001). While non-significant (*p* > 0.0016), substantial effects were observed in other analyses, whereby the odds of players attaining better perceived sleep quality was 89%, 87%, and 79% lower on congested game nights than training nights before games (OR [95% CI] = 0.11 [0.03, 0.46]), control nights (OR [95% CI] = 0.13 [0.02, 0.81]), and training nights (OR [95% CI] = 0.21 [0.02, 2.02]).

## 4. Discussion

This study adds to the limited sleep data reported in female basketball players and provides novel comparisons in sleep across different nights surrounding training and games longitudinally across the in-season. The key findings of the present study were: (1) the semi-professional, female basketball players investigated attained an adequate sleep duration (>7 h per night) and efficiency (≥85%) on average across the in-season; (2) players’ sleep was unaffected following team-based, on-court training sessions, unless training was performed the night before a game; and (3) players experienced less sleep following non-congested games, with only perceived sleep quality being further diminished when more games were played in a congested schedule. 

Overall, the findings of this study and those reported in other studies [14,33,34] suggest female basketball players attain adequate sleep durations (>7 h per night), according to the National Sleep Foundation’s recommendations for adults [2]. Indeed, semi-professional, female basketball players in the present study attained a nightly sleep duration of 7.3 ± 0.3 h (marginal mean ± SE) across all nights, which is comparable to average total sleep times reported previously in professional, female basketball players (8.1 ± 1.6 h [33], 7.6 ± 1.5 h [34], and 7.4 ± 1.5 h per night [14]). Regarding sleep efficiency, despite the values obtained by players in the present study (marginal mean ± SE across all nights = 85 ± 2%) being lower than averages reported in professional, female basketball players (92 ± 5% [33], 92 ± 4% [34], and 88 ± 6% [14]), these data were also adequate, according to recommendations from the National Sleep Foundation, bordering on the lowest cut-point for adults (≥85%) [35]. Collectively, these findings suggest female basketball players at the semi-professional (NBL1) and professional (WNBL) levels achieve adequate sleep on average across the season. However, variations in the sleep patterns of players emerged across different night types in the present study, suggesting that players may be susceptible to poorer sleep when faced with certain challenges across a competitive season [9].

A novel finding of the present study was that nights after team-based, on-court training sessions with games played on the following day had the greatest impact on the sleep patterns of players. Specifically, players exhibited significantly shorter total sleep time (*p* < 0.05) on training nights before games compared to control nights, training nights, nights before games, and congested game nights. This finding may be partially attributed to the scheduling of training sessions interacting with early morning team requirements on the following day in which games were played. Specifically, coaching staff kept consistent schedules for team-based, on-court training sessions across the season (Tuesday and Thursday evenings, Figure 1), including the night before most games played on Fridays (5 out of 6 games, Figure 1). In this way, on these game days, players were required to awaken early for travel to a new destination (away games) or participate in technical tactical sessions (home games), which is reinforced by the significantly earlier sleep offset time observed on these days compared to all other night types (Table 2 and Supplementary Table S1). In addition, although we observed adequate sleep patterns on the nights prior to games without training being completed, ruminating about an upcoming game is a predominant reason for poor sleep that athletes often experience the night before competition [52]. In this way, it is probable that scheduling training sessions the night before a game, during which discussions among players and coaching staff on tactical strategies and the upcoming opposition are likely to occur, may shift the attention and thoughts of players to promote greater rumination tendencies that impact sleep [53,54]. Moreover, upon awakening, players may have been ruminating over events, strategies, or decisions made at training during the previous night, which could have contributed to the shorter time spent in bed compared to nights before games when training was not held. Nevertheless, based on the observational findings in our study, basketball coaches may need to be cautious in scheduling team training sessions on the nights that are immediately prior to games, and consider the activities and travel arrangements closely on game day in these scenarios to ensure disruptions to sleep are avoided leading into competition. 

In the present study, players experienced poorer sleep patterns following non-congested games, including less total sleep time (*medium* effects compared to control nights, training nights, and nights before games) and later sleep onset (*medium* effects compared to control nights, training nights, training nights before games, and congested game nights). Notably, non-congested game nights in the present study consisted of home games played in isolation for that given week, as well as the first away game played within a congested game schedule. Nevertheless, this finding is likely attributed to post-game activities and rumination [52] following non-congested game nights. Indeed, following home games, post-game activities, such as showering and changing at the venue, participating in interviews, networking with sponsors, and engaging with fans, likely contributed to later bedtimes among players. Moreover, following the first away game in a congested schedule, players may be more psychologically aroused, via rumination over their performance in the game played earlier that evening, as well the upcoming game to be played on the following day [52]. While the effects observed for non-congested game nights were subdued when examining congested game nights, players were 79–89% less likely to obtain a better perceived sleep quality on congested game nights compared to control nights, training nights, training nights before games, and nights before games. While the exact mechanisms remain unknown, the lower perceived sleep quality observed on congested game nights may be due to the amplified loading experienced during congested game schedules among basketball teams [55]. In this way, two or three games played in the same week have been shown to amplify the total loading when compared to a single game week in semi-professional, male basketball players [55], which may increase the extent of muscle damage and muscle soreness experienced [9]. In this regard, muscle damage and soreness are thought to diminish perceived sleep quality in athletes [9,56]. As such, incorporating recovery techniques to reduce muscle soreness during congested game schedules may be advantageous to protect the perceived sleep quality of players across the season.

Previously, Miles et al. [14] reported a significantly (*p* < 0.05) higher wake after sleep onset on the night before games than non-congested game nights in professional, female basketball players. Although no significant (*p* > 0.05) differences between night types were observed for wake after sleep onset in the present study, *medium* effects were found, where players experienced more wake after sleep onset on nights before games than control nights, training nights before games, and non-congested game nights. These findings align with those reported by Miles et al. [14] and may be associated with players giving greater attention and thought to upcoming games, promoting disrupted sleep throughout the night [52]. Consequently, studies examining the viability of strategies to limit narrow attentional focus the night before games are encouraged, as they may lessen rumination about games and improve sleep continuity among players. Similarly, Staunton et al. [34] reported a significantly greater reduction in total sleep time on congested game nights compared to non-congested game nights in professional, female basketball players, contrasting the non-significant, *small* effects observed for this comparison in the present study. However, given that Staunton et al. [34] did not report sleep variables that indicate the underlying patterns of the players investigated (i.e., wake after sleep onset, time in bed, sleep onset, and sleep offset), it is difficult to pinpoint reasons for variations in findings across studies. Nevertheless, both studies used team-based case series designs, and therefore variations in the travel requirements, game schedules, game demands, recovery strategies, and player factors (e.g., sleep preferences, lifestyles, psychological attributes, mobile device use, sleep knowledge) across the investigated teams are possible reasons for the contrasting results. 

When interpreting the descriptive findings provided in this study, there are some notable limitations which should be considered. Firstly, sleep data were obtained in a small sample (*n* = 7) of players from a single team, and the number of samples obtained for some night types was relatively low (especially for perceived sleep quality data), meaning that differences in sleep surrounding training and games should be interpreted with caution, due to the study being underpowered. It should be noted, however, that the GLMM analyses used in this study were developed to account for an unbalanced design. Further, we reported effect sizes (i.e., Cohen’s *d* and OR) to allow for greater practical application of the data. This issue is not uncommon in applied basketball research, given that a lower number of players consistently receive adequate playing time across the season compared to other field-based team sports. Therefore, while challenging, due to geographical and resource constraints, multi-team studies through collaboration should be explored in future research on this topic for a larger sample of players to be captured. Secondly, the longitudinal and observational nature of the study meant that it was not possible to control for the menstrual cycle of players and logistical limitations (i.e., inability to conduct hormonal or urine testing, due to facility and funding constraints) negated the ability to measure this variable in the investigated players. In this way, the menstrual cycle may affect the time spent in bed and perceived sleep quality among female athletes [26,57] and should be explored in future studies. Thirdly, this study had a clear focus on sleep duration and quality surrounding training and games; however, some relevant covariates were not accounted for in our study, such as player chronotype, training and game demands, and sleep hygiene practices. As such, future research should explore how these factors may influence sleep duration and quality surrounding training sessions and games in female basketball players. Lastly, pre-season sleep data were not included in this study, given that several players were absent during this seasonal phase, due to other playing commitments. 

## 5. Conclusions

The semi-professional, female basketball players monitored longitudinally in this study attained an adequate sleep duration and efficiency on average across the in-season, in line with current recommendations. However, player sleep patterns were altered around training and games, with players being particularly susceptible to poor sleep on training nights before games and following non-congested games. In turn, perceived sleep quality diminished on nights following congested games among players. Consequently, basketball coaches should be mindful of protecting player sleep across the season when scheduling and organizing training sessions, travel, and other activities for players. In this way, avoiding team-based, on-court training sessions on nights prior to games, and adopting suitable psychological and recovery strategies around games to optimize player sleep, are encouraged, based on the present findings.

## Figures and Tables

**Table 1:** Sleep variables and associated descriptions used in the current study.

Variable (Unit)	Description
***Activity monitor***	
Sleep onset (hh:mm)	Time of day when first falling asleep.
Sleep offset (hh:mm)	Time of day when last waking before getting up.
Total sleep time (min)	Time between sleep onset and offset.
Wake after sleep onset (min)	Total amount of time spent awake between sleep onset and offset.
Sleep efficiency (%)	Total sleep time as a percentage of time in bed.
Time in bed (min)	Time between bedtime and waketime which are determined by the level of activity detected.
***Sleep diary***	
Sleep quality (AU)	Perceived quality of sleep using a 5-point Likert-type scale.

Abbreviations: AU, arbitrary units.

**Table 2:** Marginal mean ± standard error for sleep variables during different night types across the in-season in a semi-professional, female basketball team.

Sleep Variable (Unit)	Night Type							*p*
	*Overall*	*Control*	*After Training*	*After Training, before Games*	*Before Games*	*After Non-Congested Games*	*After Congested Games*	
**Total sleep time (min)**	435 ± 18	445 ± 22	445 ± 165	387 ± 15	444 ± 16	393 ± 23	424 ± 20	<0.001 *
Wake after sleep onset (min)	34 ± 6	32 ± 6	35 ± 7	29 ± 8	48 ± 15	32 ± 5	38 ± 10	0.15
Time in bed (min)	517 ± 16	522 ± 19	533 ± 15	474 ± 15	533 ± 15	482 ± 28	519 ± 29	<0.001 *
Efficiency (%)	85 ± 2	86 ± 2	84 ± 2	83 ± 3	84 ± 3	84 ± 2	82 ± 2	<0.001 *
Sleep onset (hh:mm)	23:40 ± 00:13	23:34 ± 00:17	23:38 ± 00:13	23:40 ± 00:12	23:22 ± 00:16	24:25 ± 00:18	23:43 ± 00:11	<0.001 *
Sleep offset (hh:mm)	07:27 ± 00:10	07:31 ± 00:12	07:29 ± 00:14	06:35 ± 00:09	07:30 ± 00:13	07:29 ± 00:15	07:27 ± 00:15	<0.001 *

Note: * denotes significant (*p* < 0.05) main effect detected in generalized linear mixed model for night type (reference category = control).

**Table 3:** Frequency counts (*n*) and percentages (%) of each perceived sleep quality rating given to each night type across the in-season in the semi-professional, female basketball players monitored in this study.

Night Type	Perceived Sleep Quality Rating				
	*1*	*2*	*3*	*4*	*5*
Control	*n* = 0, 0%	*n* = 8, 5%	*n* = 33, 22%	*n* = 104, 71%	*n* = 2, 1%
After training	*n* = 0, 0%	*n* = 1, 3%	*n* = 13, 37%	*n* = 20, 57%	*n* = 1, 3%
After training, before games	*n* = 0, 0%	*n* = 0, 0%	*n* = 3, 23%	*n* = 10, 77%	*n* = 0, 0%
Before games	*n* = 0, 0%	*n* = 0, 0%	*n* = 2, 25%	*n* = 6, 75%	*n* = 0, 0%
After non-congested games	*n* = 0, 0%	*n* = 4, 17%	*n* = 7, 30%	*n* = 11, 48%	*n* = 1, 4%
After congested games	*n* = 0, 0%	*n* = 3, 30%	*n* = 4, 40%	*n* = 2, 20%	*n* = 1, 10%

Note: Perceived sleep quality ratings given as: 1 = very poor sleep quality; 2 = poor sleep quality; 3 = average sleep quality; 4 = good sleep quality; and 5 = very good sleep quality.

**Table 4:** Odds ratios (OR) with 95% confidence intervals (CI) for perceived sleep quality between night types across the in-season in the semi-professional, female basketball players monitored in this study.

Model	Night Type	OR (95% CI)	*p*
Model 1	Control	*Reference*	
	After training	0.63 (0.26, 1.50)	0.29
	After training, before games	1.15 (0.47, 2.81)	0.75
	Before games	1.10 (0.34, 3.56)	0.88
	After non-congested games	0.41 (0.09, 1.82)	0.24
	After congested games	0.13 (0.02, 0.81)	0.03
Model 2	Control	1.60 (0.67, 3.83)	0.29
	After training	*Reference*	
	After training, before games	1.84 (0.65, 5.23)	0.24
	Before games	1.75 (0.33, 9.30)	0.51
	After non-congested games	0.66 (0.13, 3.22)	0.60
	After congested games	0.21 (0.02, 2.02)	0.18
Model 3	Control	0.87 (0.36, 2.11)	0.75
	After training	0.54 (0.19, 1.54)	0.25
	After training, before games	*Reference*	
	Before games	0.95 (0.36, 2.50)	0.92
	After non-congested games	0.36 (0.12, 1.06)	0.06
	After congested games	0.11 (0.03, 0.46)	0.002
Model 4	Control	0.91 (0.28, 2.96)	0.88
	After training	0.57 (0.11, 3.03)	0.51
	After training, before games	1.05 (0.40, 2.77)	0.92
	Before games	*Reference*	
	After non-congested games	0.37 (0.15, 0.93)	0.04
	After congested games	0.12 (0.05, 0.30)	<0.001 §
Model 5	Control	2.44 (0.55, 10.84)	0.24
	After training	1.53 (0.31, 7.49)	0.60
	After training, before games	2.81 (0.95, 8.37)	0.06
	Before games	2.67 (1.07, 6.68)	0.04
	After non-congested games	*Reference*	
	After congested games	0.32 (0.10, 0.98)	0.05
Model 6	Control	7.70 (1.23, 47.97)	0.03
	After training	4.81 (0.49, 46.80)	0.18
	After training, before games	8.88 (2.18, 36.21)	0.002
	Before games	8.43 (3.33, 21.37)	<0.001 §
	After non-congested games	3.15 (1.02, 9.80)	0.047
	After congested games	*Reference*	

Note: § denotes that nights after congested games were significantly associated with lower odds of attaining better perceived sleep quality than nights before games, using an alpha value adjusted with Bonferroni correction to 0.0016.

## Data Availability

All supporting data are available upon request.

## Supplementary Materials

The following supporting information can be downloaded at: https://www.mdpi.com/article/10.3390/brainsci13020238/s1, Supplementary Table S1. Pairwise comparison statistics (*p* values and Cohen’s *d* effect sizes) between night types for sleep variables.
