# Supplementary material for: Sleep Patterns Fluctuate Following Training and Games across the Season in a Semi-Professional, Female Basketball Team
Source: Brain Sci. 2023 Jan 31;13(2):238. doi: 10.3390/brainsci13020238 (PMC9954585; doi:10.3390/brainsci13020238)
Supplement: Supplementary file 1 [file brainsci-13-00238-s001.zip › brainsci-2147855-supplementary.pdf]

Supplementary Table S1. Pairwise comparison statistics (*P* values and Cohen's *d* effect sizes) between night types for sleep variables.

Sleep variable	Comparison night type	***P*, Cohen's *d***				
		Control	*After training*	*After training, before games*	*Before games*	*After non-congested games*
Total sleep time	Control	-	-	-	-	-
	After training	1.00, -0.01	-	-	-	-
	After training, before games	0.02*, -0.68‡	<0.001*, -0.68‡	-	-	-
	Before games	1.00, -0.02	1.00, 0.07	0.002*, 0.69‡	-	-
	After non-congested games	0.30, -0.61‡	0.26, -0.61‡	1.00, 0.07	0.30, -0.60‡	-
	After congested games	0.77, -0.25	0.85, -0.24	0.02*, 0.43	0.93, -0.23	0.83, 0.35
Wake after sleep onset	Control	-	-	-	-	-
	After training	0.99, 0.08	-	-	-	-
	After training, before games	0.95, -0.12	0.81, -0.20	-	-	-
	Before games	0.85, 0.52‡	0.90, 0.44	0.65, 0.67‡	-	-
	After non-congested games	1.00, 0.00	1.00, -0.08	0.99, 0.12	0.91, -0.54‡	-
	After congested games	0.99, 0.20	1.00, 0.12	0.86, 0.32	0.99, -0.32	0.93, 0.20
Time in bed	Control	-	-	-	-	-
	After training	0.93, 0.10	-	-	-	-
	After training, before games	0.70, -0.43	0.14, -0.54‡	-	-	-
	Before games	1.00, 0.10	1.00, 0.00	<0.001*, 0.54‡	-	-
	After non-congested games	0.57, -0.36	0.31, -0.47	1.00, 0.07	0.73, -0.47	-
	After congested games	1.00, -0.03	1.00, -0.13	0.88, 0.40	1.00, -0.13	0.87, 0.33
Efficiency	Control	-	-	-	-	-
	After training	0.59, -0.15	-	-	-	-
	After training, before games	0.88, -0.25	1.00, -0.10	-	-	-
	Before games	0.97, -0.22	1.00, -0.07	1.00, 0.03	-	-
	After non-congested games	0.85, -0.21	1.00, -0.06	1.00, 0.04	1.00, 0.01	-
	After congested games	0.22, -0.33	0.93, -0.18	1.00, -0.08	1.00, -0.11	0.99, -0.12
Sleep onset	Control	-	-	-	-	-
	After training	1.00, 0.05	-	-	-	-
	After training, before games	0.99, 0.08	1.00, 0.02	-	-	-
	Before games	0.98, -0.16	0.68, -0.22	0.21, -0.24	-	-
	After non-congested games	0.01*, -0.68‡	0.57, 0.63‡	0.23, 0.61‡	0.14, 0.89	-
	After congested games	0.99, 0.12	1.00, 0.07	1.00, 0.05	0.79, 0.29	0.06, -0.56‡
Sleep offset	Control	-	-	-	-	-
	After training	1.00, -0.02	-	-	-	-
	After training, before games	<0.001*, -0.76‡	0.003*, -0.76‡	-	-	-
	Before games	1.00, -0.01	1.00, 0.01	0.007*, 0.79‡	-	-
	After non-congested games	1.00, -0.02	1.00, 0.00	0.007*, 0.77‡	1.00, -0.010	-
	After congested games	1.00, -0.05	1.00, -0.03	0.002*, 0.74‡	1.00, -0.04	1.00, -0.03

Note: *denotes significant pairwise difference. ‡ denotes medium effect for pairwise difference ($d \geq 0.50$).
